# Practically Error‐Free Junctions Enable Solving Large Instances of Exact Cover Problems Using Network‐Based Biocomputation

**DOI:** 10.1002/smll.75307

**Published:** 2026-08-24

**Authors:** Eugene Christo V R, Christoph Robert Meinecke, Bert Nitzsche, Roman Lyttleton, Cordula Reuther, Danny Reuter, Heiner Linke, Till Korten, Stefan Diez

**Affiliations:** ^1^ B CUBE – Center for Molecular Bioengineering TUD Dresden University of Technology Dresden Germany; ^2^ Center for Micro and Nano Technologies Technische Universität Chemnitz Chemnitz Germany; ^3^ Fraunhofer Institute for Electronic Nano Systems ENAS Chemnitz Germany; ^4^ Cluster of Excellence Physics of Life TUD Dresden University of Technology Dresden Germany; ^5^ NanoLund and Solid‐State Physics Lund University Lund Sweden; ^6^ Helmholtz Zentrum Dresden Rossendorf HZDR Dresden Germany; ^7^ Max Planck Institute of Molecular Cell Biology and Genetics Dresden Germany

**Keywords:** biocomputation, exact cover problem, kinesin–microtubule motility, nanofabrication, NP‐complete problem, parallel computing

## Abstract

Network‐based biocomputing (NBC) presents an energy‐efficient, parallel computing approach for solving nondeterministic polynomial time (NP) complete problems by leveraging motor‐driven cytoskeletal filaments that explore all possible solutions through nanofabricated networks in a massively parallel fashion. However, guiding errors at pass junctions, where filaments deviate from their intended path, currently limit the scalability of NBC systems. In this study, we addressed this critical challenge by fabricating sub‐200 nm channel geometries using modified electron‐beam‐lithography and reactive‐ion‐etching protocols to physically constrain the trajectories of kinesin‐driven microtubules and enhance path fidelity. Investigating junction designs with varying channel widths, we demonstrate that reducing channel width significantly lowers junction error rates. Practically error‐free junction performance was achieved by scaling down the entire network geometry by a factor of two. These optimized junctions were incorporated into NBC networks that successfully solved 24‐ and 25‐set instances of the Exact Cover problem, representing solution spaces of approximately 16 and 33 million, respectively. This work establishes a new benchmark in NBC performance and represents a computational scale far beyond what has been achieved in prior demonstrations.

## Graphical abstract

Scaling nanofluidic channel geometries to sub‐200 nm is demonstrated to suppress junction guiding errors in network‐based biocomputing. The resulting increase in path fidelity enables experimental solution of Exact Cover instances with more than 33 million possible solutions, establishing a new benchmark for network‐based biocomputing.

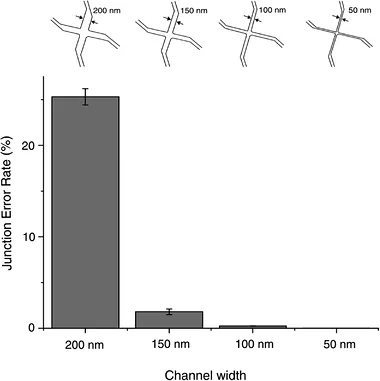

## 1 Introduction

The increasing prevalence of data‐intensive applications in areas such as operations research, molecular biology, cryptography, and artificial intelligence has emphasized the need for solving complex combinatorial problems [1, 2, 3, 4, 5]. A particularly important class of such problems is those categorized as non‐deterministic polynomial‐time complete (NP‐complete) problems. Their computational complexity grows exponentially with input size, rendering them intractable on conventional, and serial operating computers. Although heuristic and approximate algorithms can sometimes reduce computation time, they often sacrifice accuracy and cannot guarantee a solution within acceptable time frames for all instances [6, 7]. Furthermore, the stagnation of Moore's Law and the rising energy losses associated with continued CMOS miniaturization have spurred interest in exploring alternate, energy‐efficient computing approaches [8, 9, 10].

In this context, network‐based biocomputation (NBC) has emerged as a promising parallel computing strategy that leverages the nanoscale transport properties of molecular motors and cytoskeletal filaments in engineered synthetic environments [11]. Here, a combinatorial problem is physically encoded into a nanofabricated network, and kinesin‐driven microtubule filaments solve the problem by exploring all potential paths simultaneously. NBC devices require orders of magnitude less energy than traditional electronics due to the inherent energy‐efficiency of molecular motors and the fundamental energy advantage of parallel computing [12].

In this study, we use the Exact Cover problem, relevant to resource‐allocation tasks such as airline fleet planning [2] and cloud‐computing scheduling  [5], both as a benchmark problem and as an example for illustrating the operation of NBC. The Exact Cover problem consists of a target set X and a collection of subsets S. The goal is to determine whether there exists a subcollection S*⊆ S such that every element of X appears exactly once across the subsets in S*. In other words, the chosen subsets S* must be pairwise disjoint, and collectively cover all the elements of target set X without omissions or repetitions.

The implementation of Exact Cover on an NBC device (Figure 1A–C), as described in our previous works [13], involves mapping each element of the set *X* to a binary position. Each subset in *S* is similarly represented as a binary number according to this mapping, where a bit set to ‘1’ indicates the presence of the corresponding element in *X*, and a ‘0’ its absence. The numerical values assigned to the elements of *X* are arbitrary and hold no computational significance; only their mapping positions matter. For instance, whether the elements are numbered 1–6 or randomly between 115 and 12 849, the computation proceeds identically, since each element is uniquely mapped to a bit position. The number of required binary position required is solely determined by the size of the target set *X*.

**FIGURE 1:**
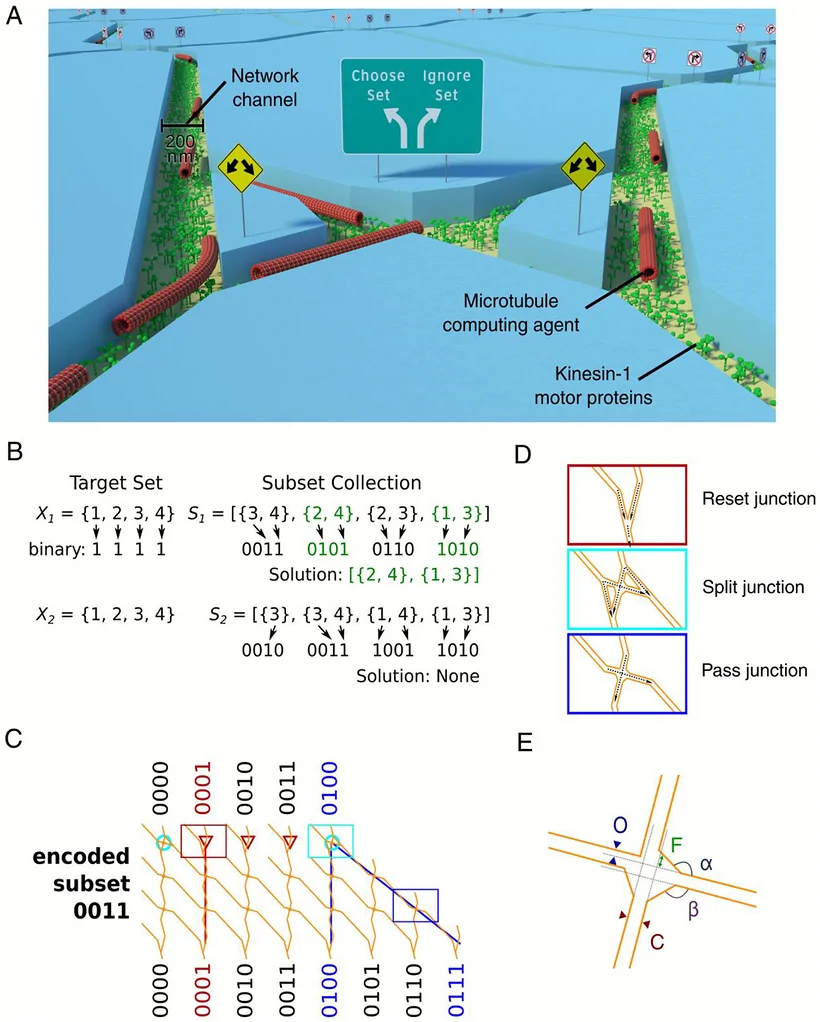
Working principle of an NBC network that encodes Exact Cover. (A) Schematic rendering of a biocomputation network. Kinesin‐1 motors coat the bottom of 200 nm channels etched in poly (methyl methacrylate) (PMMA). The channels are explored by microtubules propelled by kinesin‐1 motors. (B) Binary encoding principle: Elements of the target set X are mapped to bits of a binary number. Each subset in S is converted to a binary number by the same mapping rule. i.e., a bit is set to 1 if the respective element in X is a member of the subset and set to 0 otherwise. Two examples of exact cover instances (Xa Sa and Xb Sb) are given. Xa Sa has a solution (highlighted in green) and Xb Sb does not. (C) Example network block that encodes the subset 0011. Microtubule agents arriving at input 0100 (blue) encounter a split junction (example denoted by cyan rectangle) that allows the agents to randomly choose (diagonal path) or disregard (the path straight down) the subset encoded by this network block. If the subset is chosen, the number 0011 is added to the input 0100 and the agent arrives at the output 0111. Otherwise, the agent stays in the input column 0100. Because input subsets containing identical elements cannot be combined (as this would violate the rules of exact cover), all inputs that contain elements already present in the encoded subset start with a reset junction (example denoted by red rectangle). This junction forces the agents to take the path straight down, i.e. disregarding the encoded subset. For example, an agent entering at input 0001 (red rectangle) can only leave at output 0001. The input and output rows are separated by two rows of pass junctions (example denoted by blue rectangle) that allow agents to continue a diagonal path, incrementing the value of the binary number, or continue moving directly down and maintaining the value of the binary number. Thus, the number of pass junction rows ultimately defines which elements are contained in the encoded subset. A full network can contain many network blocks. (D) Schematic representation of each junction type. The color of the frame corresponds to the rectangles in (C). The black dotted arrows represent the permissible microtubule paths. (A–D) Adapted with permission under a Creative Commons Attribution 4.0 license from Korten et al. [13]. Copyright 2021 IOP Publishing. (E) Schematic of pass junction illustrating its geometric parameters such as channel width (denoted as C), offset (denoted as O), funnel width (denoted as F), and funnel angles (denoted as α and β). Adapted from Surendiran, P et al. [16].

Each binary subset defines a network block that guides microtubule trajectories through three distinct junction types. Microtubule agents arriving at a split junction are allowed to randomly choose (diagonal path) or disregard (the path straight down) the subset encoded by this network block. While microtubule agents encountering a reset junction are forced to take the path straight down, i.e disregarding the encoded subset. Because input subsets containing identical elements cannot be combined (as this would violate the rules of exact cover), all inputs that contain elements already present in the encoded subset encounter a reset junction. The input and output rows are separated by pass junctions that allow agents to continue a diagonal path, incrementing the value of the binary number, or continue moving directly down and maintaining the value of the binary number. The permitted path a microtubule can take in these junctions are denoted by black dotted arrows in Figure 1D. All the network blocks are concatenated to form the full computational network. The goal is for a microtubule agent to reach a final binary state where all bits are set to ‘1’, corresponding to a complete non‐redundant cover for target set *X*. The presence of such a path indicates a valid Exact Cover solution. All paths are explored simultaneously and the computational power of NBC lies in this massively parallel exploration capability. Proof‐of‐concept studies have demonstrated NBC's ability to solve small instances of NP‐complete problems, including the Subset‐Sum Problem (SSP) with eight possible solutions [11], the Exact Cover problem with 1024 possible solutions [14], and 3‐Satisfiability (3‐SAT) problems involving up to three variables and five clauses [15].

However, further scaling NBC to solve larger and more practically relevant combinatorial problems is currently hindered by the error rates associated with NBC. One principal source of error is junction errors, which occur at pass junctions when filaments deviate from their intended paths and take a wrong turn, leading to incorrect computations. As junction errors scale with network size [16, 17], they become increasingly significant for larger combinatorial problems, since the number of pass junctions a microtubule must traverse increases proportionally. Consequently, the probability that a microtubule follows the correct path across all junctions scales as (1 – e)*^J^*, where e is the junction error rate and J is the number of pass junctions traversed. For instance, with a junction error rate of approximately 2%, networks with only two pass junction rows can be reliably explored before the overall error rate for a path through the network increases above 5%. In contrast, error rates of 0.5% and 0.1% allow the use of networks with ten and fifty rows, respectively. This highlights the critical impact of even small junction error rates on the overall computational fidelity of the NBC systems. Therefore, minimizing junction errors, which is the focus of this work, is essential in order to solve large combinatorial problems of practical importance [16].

Here we demonstrate a substantial increase in the computational scale of NBC, enabled by practically error‐free operation of pass junctions. We used a stepwise approach, where we first designed a series of junction geometries with varying channel widths, deliberately using an error‐prone junction design to quantify how geometric confinement influences computational fidelity. Consistent with prior experimental and simulation studies [16, 18, 19], we found that reducing the channel width constrains filament trajectories, promotes straighter microtubule gliding, and consequently lowers junction error rates. The devices were produced using electron‐beam lithography combined with reactive‐ion etching to create sub‐200 nm channels compatible with kinesin binding and microtubule motility. Building on this, we achieved practically error‐free operation of pass junctions using an optimized geometry from prior work, along with further downscaling of the entire network. Finally, we incorporated the optimized junction design into functional NBC devices encoding 24‐set and 25‐set instances of the Exact Cover problem. These devices successfully solved problem instances comprising approximately 16 and 33 million potential solutions, respectively, establishing a new benchmark for NBC performance.

## 2 Results

### 2.1 Design and Fabrication of Junction Optimization Devices

In a previous study [16] involving actin filaments, which are more flexible and thus more prone to make errors and extensive simulations, we identified that the state‐of‐the‐art junction geometry is already optimal for microtubules with respect to the offset distance, funnel width, and funnel angles α and β (Figure 1E). This leaves the design‐channel width as the remaining parameter to be optimized. However, the state‐of‐the‐art junction error rate [14] was already so low that thousands of events would be required to reliably measure it. Therefore, we selected a junction design known to exhibit an inherently higher error rate (de‐optimized junctions) [16] as basis for our design‐channel width optimization. De‐optimized junctions had an offset distance of 0 nm, a funnel width of 66 nm, and funnel angles α = 45° and β = 30°, which were kept constant, while the design‐channel width was varied from 50 to 200 nm. Unless otherwise stated, all channel widths reported throughout the manuscript refer to the nominal design‐channel widths. The fabricated junctions (Figure S1) and actual channel widths were characterized using scanning electron microscopy (SEM) and atomic force microscopy (AFM) (Figure S2).

As a first step, de‐optimized junction designs were incorporated into four small computational networks (Figure 2A) for testing purposes. Each network consisted of a landing zone where microtubules could attach and subsequently be transported into the computation area, where they explored the networks comprised of pass and split junctions. To extend the operational lifetime of filaments within the devices, feedback loops, and rectifiers enabled continuous recycling of microtubules and ensured robust unidirectional gliding of microtubule agents.

**FIGURE 2:**
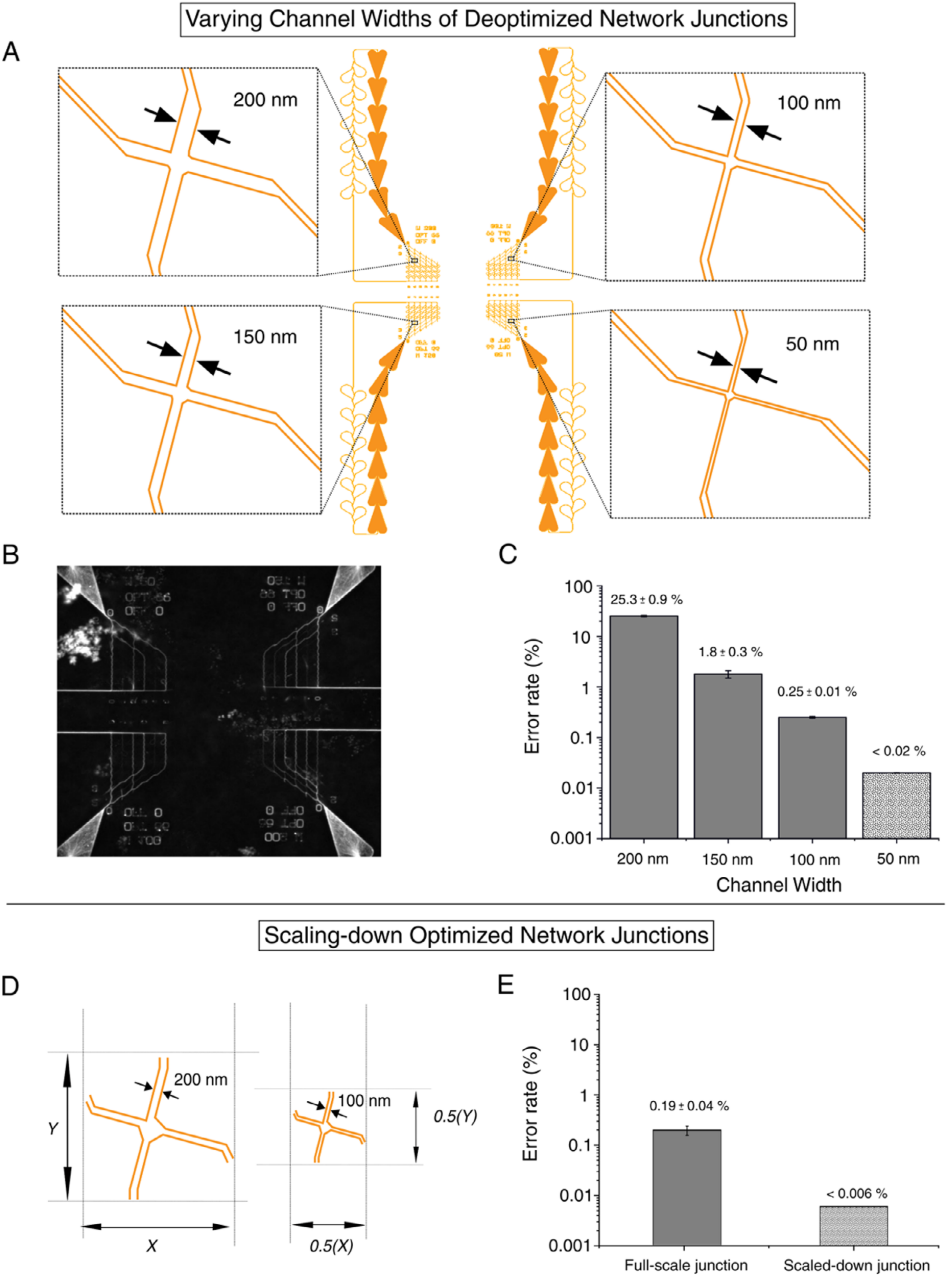
Optimization of pass junction geometry to minimize junction error rates. (A) Schematic of junction optimization networks layout showing the design of pass junctions with varying channel widths. The design‐channel widths were varied between 50 to 200 nm. Inset: 200 nm channel is shown in top left box, 150 nm channel is shown in bottom left box, 100 nm channel is shown in top right box and 50 nm channel is depicted in bottom right box. (B) maximum projection of microtubule tracks along the fabricated networks. (C) Bar plots showing the error rates with respect to the channel widths. Error bars denote the counting errors (square root of the values). (D) Schematic illustration of full‐scale and scaled‐down junction designs. (E) Bar plots showing the error rates for the full size (200 nm channels) and scaled down (100 nm channel) junctions.

To evaluate the performance of these junctions, kinesin‐coated networks were then explored by fluorescently labeled microtubules. Figure 2B shows the maximum projection of microtubule paths across the networks. All microtubule paths at pass junctions were analyzed to quantify the junction error rates (E), defined as

$$E=\frac{Number\;of\;incorrect\;paths}{Total\;number\;of\;passing\;filaments}$$

The measured error rates for de‐optimized junctions demonstrated an inverse relationship with channel width. Specifically, 200 nm channels exhibited an error rate of 25.3% ± 0.9% (mean ± standard deviation, *n* = 3,022), while 150 nm channels showed a significantly reduced error rate of 1.8% ± 0.3% (*n* = 2,548). Narrowing the channel design to 100 nm yielded an error rate of 0.25% ± 0.01% (*n* = 2,444). For the 50 nm design‐channel widths, no junction errors were observed across 5122 junction‐crossing events. Assuming a binomial process, the corresponding one‐sided 95% Clopper‐Pearson confidence interval [20] yields an upper bound of the junction error rate of e < 0.06% for 50 nm design‐channel widths (Figure 2C). However, reducing the design‐channel width from 100 to 50 nm comes with other practical drawbacks that limit scale‐up: (i) increased frequency of microtubule detachment across junctions: The 50 nm design‐width junctions showed a detachment rate of 4.2% per junction (43 out of 1023 junction crossing events), compared with 1.3% (19 out of 1456 events) for 100 nm. (ii) increased probability of microtubules getting stuck: 1.27% (13 out of 1023 events) for 50 nm design‐width compared with 0.69% (10 out of 1,456 events) for 100 nm design‐width.

Building on these findings for de‐optimized junctions, the design‐channel width improvements were applied to the optimized junctions currently used in state‐of‐the‐art NBC devices [14] to further reduce their junction error rates. For the improved design, the entire network geometry, originally comprising of 200 nm design‐channel widths, was uniformly scaled down by a factor of two in both length and width dimensions, resulting in a network with 100 nm design‐channel width (Figure 2D). The full‐scale pass junctions exhibited an error rate of 0.19 ± 0.04% (*n* = 11 878), whereas the scaled‐down optimized junctions showed no observed junction errors across 17 379 junction‐crossing events. The corresponding 95% Clopper–Pearson confidence interval gives an upper bound on the junction error rate of e < 0.017%, supporting the description of the optimized junctions as practically error‐free (Figure 2E). Because the optimized junction design had already reached practically error‐free performance at 100 nm design‐channel width, further reduction of the design‐channel width was not expected to provide a measurable improvement in computational fidelity, while coming with the drawbacks of increased microtubule detachment and probability of getting stuck (see above). A microtubule detachment rate of 4.2% for 50 nm design‐channel width would have meant losing approximately 56% of all microtubules throughout the network of 19 junction rows (including entrance and exit row) (1–0.958^19^ = 0.56), while a detachment rate of 1.3% for 100 nm design‐channel width meant losing only 22% of microtubules (1–0.987^19^ = 0.22). Similar considerations go for stuck microtubules. We therefore selected a 100 nm design channel width as a practical compromise between achieving practically error‐free junction performance and maintaining efficient network operation. These optimized junctions were subsequently incorporated into computational networks to solve 24‐set and 25‐set instances of the Exact Cover problem.

### 2.2 Encoding 24‐set and 25‐set Exact Cover Instances on to NBC Devices

Both the 24‐set and 25‐set instances of the Exact Cover problem used the same six‐element target set, X = {1, 2, 3, 4, 5, 6} but differed in their subset collections and whether a valid solution existed. The 25‐set instance included a collection, S_1_ = [{2,1}, {3,1}, {3,2,1}, {4,2}, {6,3,2}, {6,3,2,1}, {6,4}, {6,4,1}, {6,4,2}, {6,4,2,1}, {6,4,3}, {6,4,3,1}, {6,4,3,2}, {6,4,3,2,1}, {6,5}, {6,5,1}, {6,5,2}, {6,5,2,1}, {6,5,3}, {6,5,3,2}, {6,5,3,2,1}, {6,5,4,1}, {6,5,4,2,1}, {6,5,4,3,1}, {6,5,4,3,2}]. This instance was designed to have exactly one valid solution, consisting of the subsets [{3,1}, {4,2}, {6,5}] that together cover all elements of X exactly once and without repetition. In contrast, the 24‐set instance involved a modified collection, identical to S_1_ but with a critical subset {6,5} omitted: S_2_ = [{2,1}, {3,1}, {3,2,1}, {4,2}, {6,3,2}, {6,3,2,1}, {6,4}, {6,4,1}, {6,4,2}, {6,4,2,1}, {6,4,3}, {6,4,3,1}, {6,4,3,2}, {6,4,3,2,1}, {6,5,1}, {6,5,2}, {6,5,2,1}, {6,5,3}, {6,5,3,2}, {6,5,3,2,1}, {6,5,4,1}, {6,5,4,2,1}, {6,5,4,3,1}, {6,5,4,3,2}]. In this instance, no subcollection satisfied the exact cover condition and therefore no valid solution existed.

To efficiently encode these large instances while minimizing network complexity, we employed a previously developed strategy known as reverse exploration [13]. Since the target exit represents a binary string with all bits set to ‘1’, the network was split into a forward and reverse section, each encoding only half the total number of sets represented in the network. The forward network is explored from designated entrances and encodes the first portion of the subset collection, while the reverse network is explored backward from the target exit, and encodes the remaining subsets. The solution is identified when microtubules from both directions converge at an intermediate node, representing a valid subset collection that cover the target set. This approach reduces network size, lowers the number of required filaments, and shortens the time needed to find a solution. For the 25‐set instance, the exploration paths converge along the path corresponding to the correct solution subsets [{3,1}, {4,2}, {6,5}] (Figure 3A, the permitted microtubule paths indicated in blue), while for the 24‐set instance, no such convergence exist, indicating the absence of a valid solution (Figure 3B, the permitted microtubule paths indicated in blue).

**FIGURE 3:**
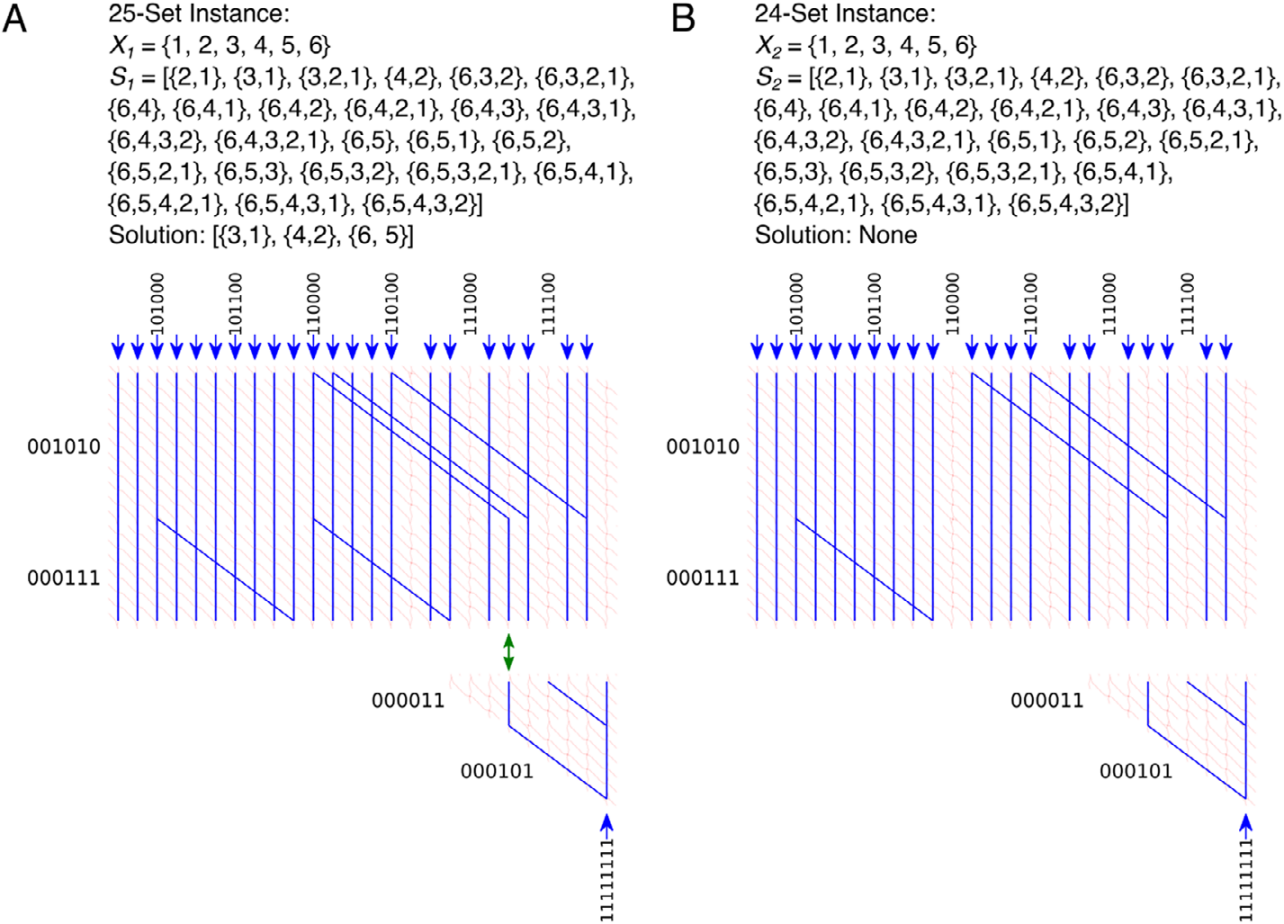
Layouts of reverse Exact Cover devices to solve Exact Cover problems with 25 and 24 set instances (33 and 16 million possible solutions). (A) Network layout of the Exact Cover instance with 25‐sets corresponding to the binary numbers: 000011, 000101, 000111, 001010, 100110, 100111, 101000, 101001, 101010, 101011, 101100, 101101, 101110, 101111, 110000, 110001, 110010, 110011, 110100, 110110, 110111, 111001, 111011, 111101, and 111110. This Exact Cover instance has a solution, which is indicated by the green double arrow. (B) Network layout of the Exact Cover instance with 24‐sets corresponding to the binary numbers: 000011, 000101, 000111, 001010, 100110, 100111, 101000, 101001, 101010, 101011, 101100, 101101, 101110, 101111, 110001, 110010, 110011, 110100, 110110, 110111, 111001, 111011, 111101, and 111110. This Exact Cover instance has no solution. (A, B) Correct paths are highlighted in blue.

### 2.3 Solving 24 and 25 Set Instances of Exact Cover Problem with NBC

The networks were explored by fluorescently labeled, kinesin‐propelled microtubules (Figure 4A,B). Filament movement was tracked using time‐lapse fluorescent microscopy with a 2‐s interval. To assess the computational output, the number of microtubule filaments reaching each network exit were statistically analyzed. The statistical significance of each outcome was determined as previously described [14] (see also Methods). The filament counts at each exit are shown in Figure 4C–F. Exits where filament counts exceeded a predefined significance threshold (*p* < 0.05, green dash‐dotted lines) were classified as correct exits (shown in green) Exits below a corresponding lower threshold (magenta dotted lines) were classified as incorrect (shown in red).

**FIGURE 4:**
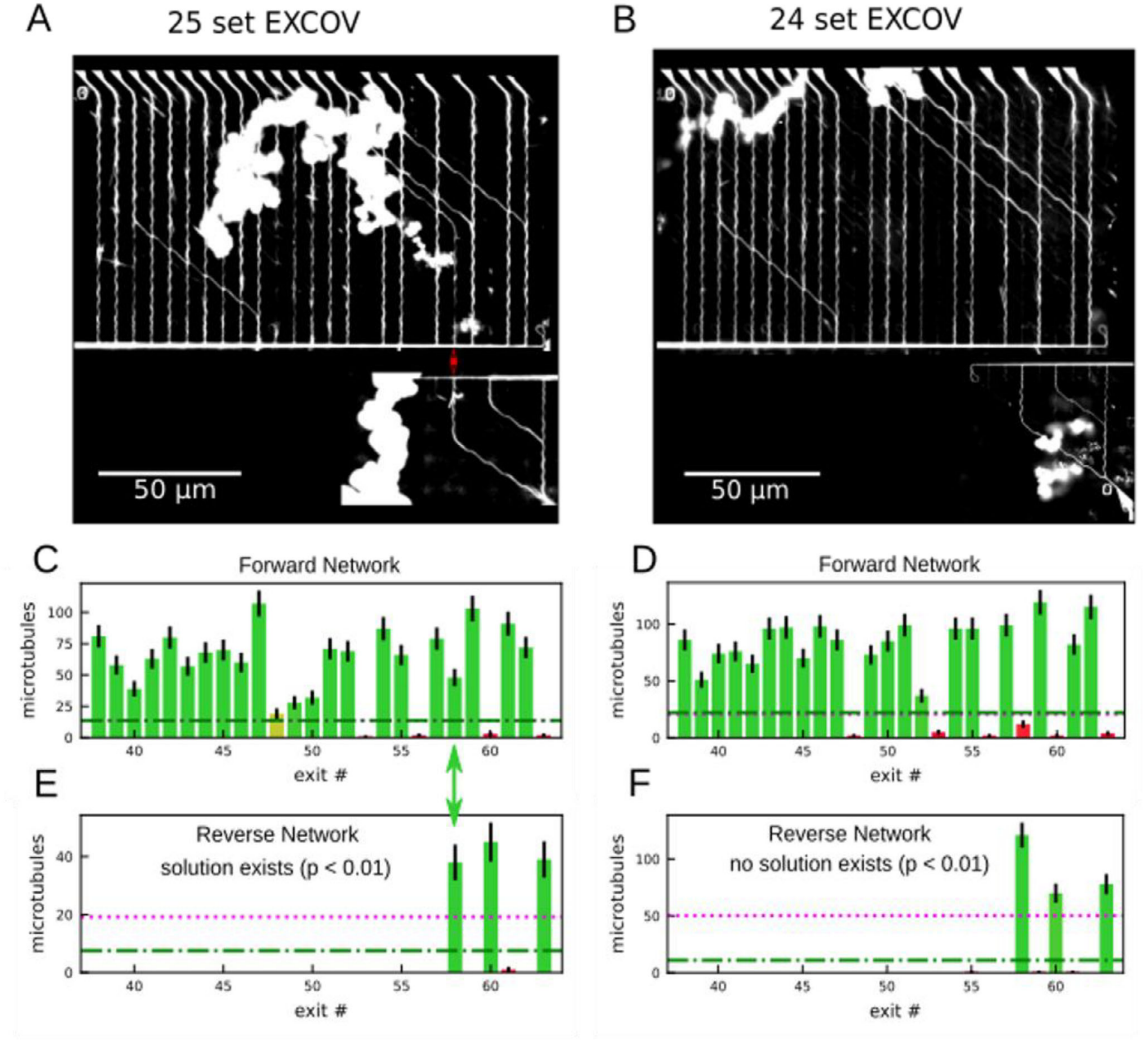
Microtubule results for the 25‐set and 24‐set Exact Cover instances: (A, C, E) 25‐set instance with exactly one solution (indicated by green double arrow in C and E). (B, D, F) 24‐set instance without solution. (A, B) Standard deviation plots of 600 frames of 2 s time‐lapse fluorescence micrographs of microtubules moving through the computational network. Paths that were more frequently visited by microtubules are brighter. (C–F) Number of microtubules counted at each exit of the (C, D) forward‐ and (E, F) reverse halves of the Exact Cover networks. RGB colors indicate probabilities that the respective counts correspond to a correct (more green) or an incorrect (more red) exit. Error bars represent the counting error (square root of the respective value). Values above the green dash‐dotted lines are significant correct exits (*p* < 0.05), values below the magenta dotted lines are significant incorrect exits (*p* < 0.05). In total, the calculation took 20 min for each network.

During NBC operation, no pass junction errors were observed across 17 379 events, confirming the reliability of the optimized junction design. This practically error‐free performance enabled microtubules to traverse the networks with an overall path success probability exceeding 99.9%. Given that each microtubule traverses 17 pass junctions, even a single error (error rate ∼ 0.000057) would reduce the overall path success probability to approximately 99.9% ((1−0.000057) [17]). Since, no such errors were observed, the actual path success probability is even higher.

Despite this high fidelity of pass junctions, a few microtubule filaments exited the network incorrectly due to landing errors, where microtubules landed at unintended positions within the network rather than through the designated entrances. Out of 2667 total exit events, 127 events were attributed to such errors. These included some filaments that still exited at correct outputs and others that exited at unintended ones. Nevertheless, the most frequently traveled microtubule paths closely matched the prediction by the network design for valid solutions (Figure 4A,B).

For the 25‐set instance, microtubules from the forward and reverse networks successfully converged at the correct solution path at exit point 111010, confirming the existence and successful identification of a valid Exact Cover (*p* < 0.01; significance was determined as described in [14]). This solution followed a complete path starting at subset {5,6}, corresponding to entrance 110000, proceeding through subset {2,4} (001010) in the forward network and through subset {1,3} (000101) in the reverse network, forming a path to the target set {1,2,3,4,5,6} (111111). In contrast, the 24‐subset instance showed no such convergence between forward and reverse paths, correctly indicating that no solution exists for that Exact Cover instance (*p* < 0.01).

While narrower channels effectively reduced junction errors, they raised the question of whether reduced kinesin density in narrower channels might increase microtubule detachment at network junctions. To assess this, detachment rates were experimentally compared between networks fabricated with a design‐widths of 100 and 200 nm channels (full‐scaled and scaled down 25‐set Exact Cover networks). No significant difference in the detachment rate per junction was observed between the two networks. In the full‐scale device, 10 out of 42 microtubules filaments detached before crossing 17 pass junctions, while 11 out of 55 microtubule filaments detached before crossing 17 pass junctions in the scaled down device (Figure S3).

The percentage of microtubules traversing each junction as a function of the number of junctions traversed was fitted using linear regression, assuming a first‐order approximation of the exponential decay function, for both networks. The slope for the full‐scale network was – 1.27 ± 0.11% per junction, and for the scaled‐down network was – 1.41 ± 0.11% per junction. Both networks showed significant negative correlations between junction number and detachment rate. Statistical comparison using an interaction model revealed no significant difference between slopes (*p* = 0.372), indicating similar detachment kinetics across network scales (Figure 5).

**FIGURE 5:**
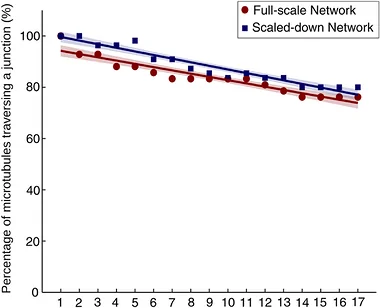
Detachment rate of microtubules per junction. Percentage of microtubules traversing each junction as a function of the number of junctions crossed in the full‐scale network (red circles) and scaled‐down network (blue squares). Lines represent linear regression fits for each condition, with shaded areas indicating 95% confidence intervals. The slope for the full‐scale network was −1.27% ± 0.11% per junction, and for the scaled‐down network was −1.41% ± 0.11% per junction. Both networks show significant negative correlations between junction number and detachment rate. Statistical comparison using an interaction model revealed no significant difference between slopes (*p* = 0.372), indicating similar detachment kinetics in both networks.

These experiments demonstrate the successful design and implementation of NBC devices capable of solving larger instances of the Exact Cover problem, specifically, a 25‐set instance (with a known solution and a sample space of approximately 33 million) and a 24‐set instance (with no solution and a sample space of approximately 16 million). The integration of reverse exploration, binary encoding, and practically error‐free junctions enables NBC systems to explore these vast combinatorial spaces in parallel with high efficiency and accuracy. Solving these problem instances marks a significant advancement in molecular computing, representing a new benchmark for NBC based computing.

## 3 Discussion

In this study, we addressed junction errors, a key limitation hindering the scalability of NBC systems. These errors become increasingly significant as network complexity increases, since the number of junctions a microtubule must traverse and thus the probability of junction error, grows with problem size. To minimize these errors, we aimed to physically constrain filament trajectories and improve path fidelity. Toward this end, a modified electron beam lithography and optimized reactive ion etching protocol was used to fabricate sub‐200‐nm channel geometries compatible with microtubule gliding assays. We then used junction designs known to exhibit high error rates to demonstrate that reducing channel width significantly lowers junction errors. Narrower channels increase the angular deviation required for a gliding microtubule to make an unintended turn, effectively preventing such errors (Figure 6).

**FIGURE 6:**
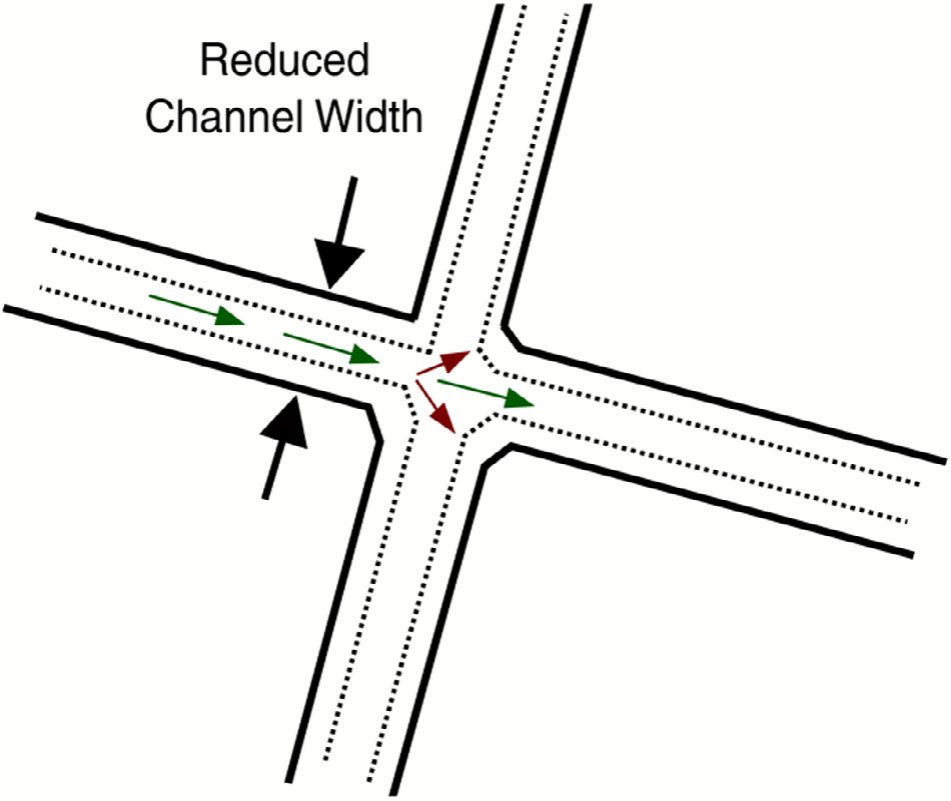
Schematic of microtubule path in a pass junction. Movement of the microtubule in the correct path is represented by green arrows. The red arrows represent a wrong turn. The black arrows show the reduced width of the channel, represented by the dotted lines. Microtubules have to turn along steeper angles to make a wrong turn in narrower channels.

The improved fabrication technology enabled us to scale down the state‐of‐the‐art junction design, which exhibited an error rate of 0.19%. In the scaled‐down networks, we did not observe a single error across 17 379 junction‐crossing events. Unlike three‐dimensional junctions with overpasses [17], the design used here does not eliminate errors in principle; however, in practice, all pass junctions operated error‐free. Importantly, this design is considerably simpler to fabricate, relying on established lithography methods. The reduction in fabrication steps not only streamlined production but also improved junction smoothness by removing residual PMMA monolayers from the channel floor, thereby enhancing performance. In addition to improving fidelity, down‐scaling reduced microtubule exploration distances, and contributing to faster computation. The low junction error observed in our experiments is highly encouraging for further scaling of NBC devices. The probability that a microtubule traverses a network containing J pass‐junction rows without a junction error is p = (1 − e)^J^, where e is the per‐junction error rate. Given the 95% confidence upper bound of the junction error rate determined experimentally (e < 0.017%), and requiring an overall probability of at least 95% to traverse the network without pass junction error, we estimate that networks containing up to at least 298 pass‐junction rows can be operated reliably. This estimate illustrates how improvements in local junction performance directly translate into the ability to construct substantially larger computational networks before junction errors become limiting.

The presented results established a new benchmark for NBC and represent a computational scale beyond what has been achieved by state‐of‐the‐art DNA computing, which to date has solved a 20‐variable 3‐SAT problem [21]. While the DNA computing technique exhaustively explored the full solution space of over one million truth assignments, it also required extensive processing steps, including sequential capture‐and‐release across 24 clause modules, followed by PCR amplification and gel‐based readout to isolate and identify the correct solution. In contrast, NBC devices in this work solved Exact Cover instances with solution spaces on the order of 16 to 33 million possibilities, an order of magnitude larger than the DNA computing study. This scaling is achieved through the use of optimization strategies such as bit mapping optimization, entrance optimization, and reverse exploration (see Korten et al. 2021 for details of the optimization algorithm [13]), which reduce network complexity while still guaranteeing correct solution identification. This difference in approach allows NBC to reach far larger problem spaces while maintaining practical device sizes and operational simplicity, by reducing the solution space that needs to be exhaustively explored. Although larger problems in NBC require a proportionally greater number of microtubules, this constraint can be mitigated by the ability to continuously supply microtubules during computation. Furthermore, producing large numbers of microtubules is far cheaper, because they self‐assemble, allowing advances such as on‐chip microtubule multiplication [22], which provide a means to sustain agent numbers during computation, further supporting scalability.

While with the optimized junctions, for an overall success of 95%, networks sizes with at least 298 pass junction rows can be solved in theory, however, in practice, this is currently limited by landing errors. These errors originated from microtubule landing within the computational network at unintended positions. In our experiments, despite the practically error‐free junction operation, microtubules were still observed at incorrect exits while solving the 24‐set and 25‐set instances of the Exact Cover problem. 127 out of 2667 filaments exhibited such behavior, which are attributed to landing errors. Although these errors did not significantly affect identification of the correct solution, they highlight the importance of controlling filament loading in future systems. To address this, we propose integrating NBC devices with microfluidic systems. Flow‐focusing can confine microtubules to specific loading zones, steering them away from computation regions, making this a viable strategy for minimizing landing errors and improving system scalability [17]. Moreover, achieving a level of parallelism that allows NBC to outperform conventional electronics in time‐to‐solution will require further progress in key areas such as programmability, scalability, and readout technology [23, 24, 25]. Such techniques would remove the reliance on bulky, high‐resolution imaging systems, and enable deployment of denser, complex, reusable, and programmable NBC networks capable of solving multiple problem instances.

In summary, this study demonstrates a significant advancement in scalability of network‐based biocomputation. By practically eliminating junction errors and validating the solution of large‐scale problem instances representing over 30 million solutions we establish a new benchmark for molecular computing. Continued development in microfluidic integration, reconfigurable architectures, compact encoding strategies, and electrical readout platforms will be essential to realizing NBC's potential as a viable, high‐performance computing paradigm.

## 4 Methods

### 4.1 Materials

All reagents were purchased from Sigma–Aldrich unless otherwise specified.

### 4.2 Microtubule and Kinesin Preparation

Microtubules were polymerized from porcine brain tubulin (purified using established protocols [26]), labeled with rhodamine in BRB80 buffer (80 mm PIPES, 1 mm EGTA, 1 mm MgCl_2_, pH 6.9), supplemented with 5 mm MgCl_2_, 1 mm MgGTP, and 5% DMSO. Polymerization was carried out at 37°C for 1 h using a 4 mg/mL tubulin mixture. The resulting microtubules were stabilized with 10 mm Taxol in BRB80 buffer and stored at room temperature. All experiments were conducted within 24 h of polymerization. Full‐length Drosophila melanogaster kinesin‐1 motor proteins were expressed in insect cells and purified as described previously [27].

### 4.3 Fabrication and Assembly of Network‐Based Biocomputing Devices

NBC networks were patterned on polymethyl methacrylate (PMMA)‐coated 150 mm highly p‐doped silicon wafers with a 75 nm silicon oxide layer. The substrates were cleaned using a standard RCA protocol to remove organic and metallic contaminants: A 1:1:5 mixture of NH_4_OH, H_2_O_2_, and deionized water (SC‐1) followed by a 1:1:6 mixture of HCl, H_2_O_2_, and deionized water (SC‐2). Both steps were conducted at 75°C for 2 min. A 75 nm thermal SiO_2_ layer was then grown at 1000°C in an oxygen and HCl‐rich atmosphere, followed by annealing at 700°C under nitrogen for 30 min to improve oxide quality. A post‐annealing step at 200°C for 30 min was performed to enhance PMMA adhesion.

Electron‐beam resist (PMMA, Allresist AR‐P 679.04) was spin‐coated at 1700 rpm to achieve a thickness of ∼440 nm and hard‐baked at 180°C for 5 min. Devices fabrication proceeded in two steps (see Figure S2): First, structures were patterned on PMMA using an VISTEC SB25, VISTEC Electron Beam GmbH, Jena, Germany operated at an accelerating voltage of 50 kV, an exposure dose of 650 µC/cm^2^. The electron beam (variable shaped beam) was raster‐scanned according to the designed pattern using design in gds format generated by layout editor/K‐Layout and transferred using ePLACE to machine compatible data. The patterned structures were then developed in a 1:3 mixture of MIBK and isopropanol for 60 s at room temperature, and rinsed with isopropanol and deionized water, and dried. Second, residual PMMA monolayers were removed from the channel floor by reactive ion etching (RIE) with CF4 plasma (20 sccm flow, 80 W RF power, 15‐s exposure). Wafers were then cleaved into 10 mm × 10 mm chips for experimental use.

### 4.4 Characterization of NBC Devices

*Scanning electron microscopy (SEM)* was conducted using a GAIA3 (TESCAN, Czech Republic) operated at an accelerating voltage of 5.0 kV and a working distance in the range of 9.93–10.02 mm. An InBeam SE detector was employed to obtain high‐resolution images. Samples were directly imaged without sputter‐coating any conductive layer.

*Atomic force microscopy (AFM)* was performed in non‐contact mode using a TI950 Nanoindenter platform (Hysitron/Bruker, USA). Scans were acquired over an area of 3 × 3 µm^2^ with a resolution of 512 × 512 pixels using a cantilever with a nominal tip radius of 7 nm. Topographic data were processed and analyzed using Gwyddion software.

SEM imaging was used to characterize the junction geometries and to assess the reproducibility of the fabrication process. For the latter, channel widths were measured from SEM images of networks fabricated in three independent fabrication batches. For the 100 nm design‐channel width one wafer from one batch containing several networks was analyzed. The measured channel width for two different network layouts was 126 ± 6 nm (mean ± SD, *n* = 6). The investigations on the 200 nm design‐channel width were performed on two wafers from two different batches. The measured channel width was 222 ± 9 nm (mean ± SD, *n* = 9). These measurements demonstrate that identical network layouts fabricated on different wafers at different times consistently produce comparable channel dimensions, confirming the robustness, and reproducibility of the fabrication process. While SEM imaging can result in widening the channels due to electron‐beam‐induced deformation/shrinkage of the PMMA, it is still reliable for comparing channel withs between fabrication batches because all sample were imaged under the same conditions. However, to determine absolute channel widths AFM imaging was used (Figure S2).

### 4.5 Experimental Validation of NBC Devices

Flow cells were assembled by placing two parallel strips of parafilm (∼2 mm apart) on either side of the patterned region on the fabricated chips, followed by placement of a 22 × 22 mm^2^ Diphenyldimethoxysilane (DDS)‐coated coverslip on top. The assembly was sealed by gentle heating for ∼20 s, producing a leak‐proof channel. The assembled flow cells were perfused with Pluronic F127 in BRB80 buffer and allowed to incubate for 2 h at room temperature to selectively passivate the PMMA surface, while leaving the SiO_2_ channel floor exposed for kinesin binding [14]. Following this, the flow cell was perfused with 0.5 mg/mL casein in BRB80 buffer and incubated for 5 min. Subsequently, 5 µL of 4 nm kinesin‐1 solution was introduced and incubated for 15 min to allow motor protein attachment. Finally, a motility buffer containing 1 mm Mg‐ATP, 20 mm D‐glucose, 20 µg/mL glucose oxidase, 10 µg/mL catalase, 10 mm DTT, and 10 µm Taxol in BRB80 was introduced, followed by rhodamine‐labeled, taxol‐stabilized microtubules.

### 4.6 Image Acquisition

Gliding motility assays were imaged using a Nikon Eclipse Ni upright fluorescence microscope equipped with a CFI S Plan Fluor ELWD 20X long‐distance, wide‐field air objective and an ORCA‐Fusion 14440 camera. Images were acquired and processed with NIS‐Elements software (Nikon). CoolLED pE4000 was used as the light source, and a multiband filter (DAPI/FITC/Cy3/Cy5/Cy7 Penta‐LED HC Filter, Semrock) was used to visualize the rhodamine‐labeled microtubules. Exposure time was set to 500 milliseconds, and time‐lapse images were recorded at 2‐s intervals.

### 4.7 Data Analysis and Statistical Methods

To evaluate the reliability of the solution in NBC, both landing errors and junction errors were quantitatively assessed. Landing errors were estimated using the equation:

L=l×J

where *l* is the landing rate per junction, and 𝐽 is the number of junctions.

The junction error rate, E was defined as:

$$E=\frac{Number\;of\;incorrect\;paths}{Total\;number\;of\;passing\;filaments}$$

The probability that a microtubule agent follows the correct path across *X* junctions scales as (1 – *E*) *x*. For experiments in which no junction errors were observed, the upper bound of the junction error rate was estimated using the 95% Clopper‐Pearson confidence interval for a binomial distribution [20]. Junction error rates are reported as percentages in the Results and Discussion to represent junction performance.

To evaluate whether the filament distribution at the network exits reliably indicated a correct solution (e.g., an exact cover), a four‐step statistical framework based on our previous work was employed [14]. This approach integrates empirical error estimates with statistical modeling to determine whether observed filament counts could be attributed to accurate computation rather than random fluctuations. Total filament counts affected by guidance errors were estimated by combining experimentally measured junction error rates with landing error estimates from Monte Carlo simulations. These data were normalized relative to the number of network exits, enabling robust confidence assessments to distinguish between correct and wrong exits.

In order to be able to handle the large amount of junction crossing events, we trained a time‐aware convolutional neural network (CNN) with Conv2DLSTM layers. Code [28], model weights, and training data [29] are available. The network was trained to classify video sequences of each junction into four classes: (0) left to right, (1) top to bottom, (2) left to bottom, (3) top to right. For a pass junction classes 0 and 1 are correct and 2 and 3 represent errors. To avoid bias, we trained the network with an equal number of events for each class. Training data consisted of 922 video sequences of shape time x height x width with 13 to 64 (mean 27, total 25 087) frames of 32 × 32 pixels each. Each video sequence was centered on one individual junction and contained at least one crossing event (total 1408 events, mean 1.5 events per sequence). Events were classified at the last frame before the microtubule tip left the video. The dataset was split into 553 video sequences for training and 367 sequences for validation. In order to improve generalization performance, training data was randomly augmented by scaling to 16 × 16, 24 × 24, or 32 × 32 pixels, rotating by up to 2 degrees clockwise and counterclockwise, and adding random amounts of noise. We trained an ensemble of 6 networks, obtaining a mean ± standard deviation accuracy of 0.772 ± 0.004. Notably, the vast majority of errors was in the temporal domain, because the network was off by one frame. Misclassification of event type was negligible. Since the timing of the events is irrelevant to this work, the network performance was adequate for our purposes. However, since the junction crossing error was extremely low, we manually re‐evaluated junction crossing errors and discarded errors that were due to the network misclassifying events when multiple microtubules crossed the junction at the same time. To qualitatively test the performance of the network on unseen data, we compared the results to manually evaluated data obtained from a previous publication [14], which was not included in the training data. The data evaluated by our network was not significantly different from the manually evaluated data.

The maximum projection images of frequented microtubules paths were made using Fiji ImageJ software, and the plots were plotted using Origin Pro 2024b. Linear regression and statistical analysis were performed in MATLAB.

## Author Contributions

D.R., H.L., T.K., and S.D. conceived the study. T.K. designed and C.R.M. nanofabricated the computation networks. E.C.V.R. and T.K. performed microtubule−kinesin experiments and analyzed the respective data. B.N., R.L., and C.R. contributed to testing and optimizing microtubule motility in the nano‐channels. E.C.V.R., T.K., and S.D. wrote the manuscript with contributions from the other authors. All authors discussed the results and approved the final version of this manuscript.

## Conflicts of Interest

The authors declare no conflicts of interest.

## Supporting information




**Supporting File**: smll75307‐sup‐0001‐SuppMat.docx.

## Data Availability

The data that support the findings of this study are available from the corresponding author upon reasonable request.

## References

[1] C. L. Philip Chen and C.‐Y. Zhang , “Data‐Intensive Applications, Challenges, Techniques and Technologies: A Survey on Big Data,” Information Sciences 275 (2014): 314–347, 10.1016/j.ins.2014.01.015.

[2] M. Grönkvist and J. Kjerrström , “Tail Assignment in Practice,” in Operations Research Proceedings 2004, eds.: H. Fleuren , D. Hertog and P. Kort (Springer, 2005), 166–173, 10.1007/3-540-27679-3_21.

[3] S. Held , B. Korte , D. Rautenbach , and J. Vygen , “Combinatorial Optimization in VLSI Design,” Combinatorial Optimization (IOS Press, 2011), 33–96, 10.3233/978-1-60750-718-5-33.

[4] F. Peres and M. C. O. P. Castelli , “The Challenges of Industry 4.0: A Review,” Applied Science 11, no. 14 (2021): 6449, 10.3390/app11146449.

[5] C. Ba , “An Exact Cover‐Based Approach for Service Composition,” in 2016 IEEE International Conference on Web Services (IEEE, 2016), 631–636, 10.1109/ICWS.2016.87.

[6] N. A. Pierce and E. Winfree , “Protein Design Is NP‐Hard,” Protein Engineering, Design and Selection 15, no. 10 (2002): 779–782, 10.1093/protein/15.10.779. 12468711

[7] W. Dong , Z.‐H. Xu , X.‐H. Liu , L.‐S.‐B. Wang , and S.‐P. Xiao , “Irregular Subarray Tiling via Heuristic Iterative Convex Relaxation Programming,” IEEE Transactions on Antennas and Propagation 68 (2020): 2842–2852, https://ieeexplore.ieee.org/document/8917927.

[8] G. E. Moore , “Cramming More Components Onto Integrated Circuits,” IEEE Solid‐State Circuits Society Newsletter 38, no. 3 (1965): 33–35, 10.1109/N-SSC.2006.4785860.

[9] T. N. Theis and H.‐S. P. Wong , “The End of Moore's Law: A New Beginning for Information Technology,” Computing in Science & Engineering 19, no. 2 (2017): 41–50, 10.1109/MCSE.2017.29.

[10] M. M. Waldrop , “The Chips Are Down for Moore's Law,” Nature 530, no. 7589 (2016): 144–147, 10.1038/530144a. 26863965

[11] D. V. Nicolau , M. Lard , T. Korten , et al., “Parallel Computation With Molecular‐Motor‐Propelled Agents in Nanofabricated Networks,” Proceedings of the National Academy of Sciences 113, no. 10 (2016): 2591–2596, 10.1073/pnas.1510825113. PMC4791004 26903637

[12] M. Konopik , T. Korten , E. Lutz , and H. Linke , “Fundamental Energy Cost of Finite‐Time Parallelizable Computing,” Nature Communications 14, no. 1 (2023): 447, 10.1038/s41467-023-36020-2. PMC9883481 36707510

[13] T. Korten , S. Diez , H. Linke , D. V. Nicolau , and H. Kugler , “Design of Network‐Based Biocomputation Circuits for the Exact Cover Problem,” New Journal of Physics 23, no. 8 (2021): 085004, 10.1088/1367-2630/ac175d.

[14] P. Surendiran , C. R. Meinecke , and A. Salhotra , “Solving Exact Cover Instances with Molecular‐Motor‐Powered Network‐Based Biocomputation,” ACS Nanoscience Au 2 (2022): 396–403, 10.1021/acsnanoscienceau.2c00013. PMC9585575 36281252

[15] J. Zhu , A. Salhotra , C. R. Meinecke , et al., “Solving the 3‐Satisfiability Problem Using Network‐Based Biocomputation,” Advanced Intelligent Systems 4, no. 12 (2022): 2200202, 10.1002/aisy.202200202.

[16] P. Surendiran , M. Ušaj , T. Korten , A. Månsson , and H. Linke , “Error‐Rate Reduction in Network‐Based Biocomputation,” arXiv (2024), arXiv:2407.01754, 10.48550/arXiv.2407.01754.

[17] F. C. M. J. M. van Delft , A. Månsson , H. Kugler , et al., “Roadmap for Network‐Based Biocomputation,” Nano Futures 6, no. 3 (2022): 032002, 10.1088/2399-1984/ac7d81.

[18] J. Clemmens , H. Hess , J. Howard , and V. Vogel , “Analysis of Microtubule Guidance in Open Microfabricated Channels Coated With the Motor Protein Kinesin,” Langmuir 19, no. 5 (2003): 1738–1744, 10.1021/la026155x.

[19] M. G. L. van den Heuvel , C. T. Butcher , R. M. M. Smeets , S. Diez , and C. Dekker , “High Rectifying Efficiencies of Microtubule Motility on Kinesin‐Coated Gold Nanostructures,” Nano Letters 5, no. 6 (2005): 1117–1122, 10.1021/nl0506554. 15943453

[20] C. J. Clopper and E. S. Pearson , “The Use of Confidence or Fiducial Limits Illustrated in THE Case of THE Binomial,” Biometrika 26, no. 4 (1934): 404–413, 10.1093/biomet/26.4.404.

[21] R. S. Braich , N. Chelyapov , C. Johnson , P. W. K. Rothemund , and L. Adleman , “Solution of a 20‐Variable 3‐SAT Problem on a DNA Computer,” Science 296, no. 5567 (2002): 499–502, 10.1126/science.1069528. 11896237

[22] C. Reuther , P. Santos‐Otte , R. Grover , G. Heldt , G. Woehlke , and S. Diez , “Multiplication of Motor‐Driven Microtubules for Nanotechnological Applications,” Nano Letters 22 (2022): 926–934, https://pubs.acs.org/doi/10.1021/acs.nanolett.1c03619. 10.1021/acs.nanolett.1c03619 35050639

[23] V. Schroeder , T. Korten , H. Linke , S. Diez , and I. Maximov , “Dynamic Guiding of Motor‐Driven Microtubules on Electrically Heated, Smart Polymer Tracks,” Nano Letters 13, no. 7 (2013): 3434–3438, 10.1021/nl402004s. 23750886

[24] R. Catalano , Y. Zhao , M. Pecak , T. Korten , and S. Diez , “Barcoding Microtubules: Encoding Information Onto Macromolecules by Photobleaching,” Nano Letters 25, no. 13 (2025): 5283–5290, 10.1021/acs.nanolett.5c00105. PMC11969655 40117582

[25] M. S. Miranda , R. Lyttleton , P. H. Siu , S. Diez , H. Linke , and A. P. Micolich , “Prospects for Single‐Molecule Electrostatic Detection in Molecular Motor Gliding Motility Assays,” New Journal of Physics 23, no. 6 (2021): 065003, 10.1088/1367-2630/abfdf5.

[26] M. Castoldi and A. V. Popov , “Purification of Brain Tubulin Through Two Cycles of Polymerization–Depolymerization in a High‐Molarity Buffer,” Protein Expression and Purification 32, no. 1 (2003): 83–88, 10.1016/S1046-5928(03)00218-3. 14680943

[27] T. Korten , S. Chaudhuri , E. Tavkin , M. Braun , and S. Diez , “Kinesin‐1 Expressed in Insect Cells Improves Microtubule in Vitro Gliding Performance, Long‐Term Stability and Guiding Efficiency in Nanostructures,” IEEE Transactions on NanoBioscience 15, no. 1 (2016): 62–69, 10.1109/TNB.2016.2520832. 26886999

[28] T. Korten , “Vidtrain,” GitLab, accessed November 25 (2025), https://gitlab.com/Thawn/vidtrain.

[29] T. Korten , “Data for Vidtrain,” Zenodo, accessed November 25 (2025), 10.5281/zenodo.17643846.

