# Supplementary material for: Practically Error‐Free Junctions Enable Solving Large Instances of Exact Cover Problems Using Network‐Based Biocomputation
Source: Small. 2026 Aug 24;22(59):e75307. doi: 10.1002/smll.75307 (PMC13690612; doi:10.1002/smll.75307)
Supplement: Supplementary file 1 [file SMLL-22-e75307-s001.docx]

Practically Error-Free Junctions Enable Solving Large Instances of Exact Cover Problems Using Network-Based Biocomputation

**Supplementary Figures**

**Figure S1**: Schematic of modified fabrication protocol for patterning sub-200 nm PMMA structures. (**A**) PMMA coated Silicon/SiO_2_ substrate was patterned with electron beam lithography. PMMA layer is shown in yellow, SiO2 layer is shown in green and silicon substrate is shown in brown. Following electron beam exposure, the structures are developed with wet etching. (**B**) The developed structures are subjected to reactive ion etching in the presence of CF_4_ plasma. This is followed by blocking the PMMA walls with Pluronic F127. Blocking the PMMA surface with Pluronic F127 prevents kinesin from binding to the PMMA surfaces and selectively bind to the SiO_2_ surface on the channel floor.

**Figure S2**: Physical characterization of junction geometries with varying widths. Scanning electron microscopy (SEM) and atomic force microscopy (AFM) analysis of pass junction geometries with varying design-channel widths. (**A**) 200 nm, (**B**) 150 nm, (**C**) 100 nm, and (**D**) 50 nm. Left panels (SEM): Low- and high-magnification images show the overall layout of the fabricated networks (green insets) and individual junctions (red insets). Right panels (AFM): Topographic maps (Scale bar = 1 µm) and corresponding cross-sectional profiles confirm the fabricated channel dimensions. For junctions with design-channel widths of 200, 150, 100 and 50 nm, the fabricated channel widths measured by AFM were 231 ± 16 nm, 170 ± 6 nm, 117 ± 9 nm and 71 ± 8 nm (mean ± standard deviation, n = 5), respectively.

**Figure S3:** Detachment rate of microtubules per junction. (**A**) Bar plots showing the number of microtubules crossing each junction and the number of junctions traversed on the full-scale NBC with 200 nm channels. 10 out of 42 microtubules filaments detached before crossing 17 pass junctions. Error bars denote counting errors. (**B**) Bar plots showing the number of microtubules crossing each junction and the number of junctions traversed on the scaled-down NBC with 100 nm channels. 11 out of 55 microtubule filaments detached before crossing 17 pass junctions. Error bars denote counting errors.
